# Glymphatic dysfunction exacerbates cognitive decline by triggering cortical degeneration in Parkinson's disease: evidence from diffusion-tensor MRI

**DOI:** 10.1093/braincomms/fcaf029

**Published:** 2025-02-20

**Authors:** Yang Zhao, Changyuan Xu, Yufan Chen, Tao Gong, Mengyuan Zhuo, Cheng Zhao, Zhanfang Sun, Weibo Chen, Yuanyuan Xiang, Guangbin Wang

**Affiliations:** Department of Radiology, Shandong Provincial Hospital Affiliated to Shandong First Medical University, Jinan 250021, China; Department of Medical Imaging Center, Central Hospital Affiliated to Shandong First Medical University, Jinan 250013, China; Department of Radiology, Shandong Provincial Hospital Affiliated to Shandong First Medical University, Jinan 250021, China; Department of Radiology, Shandong Provincial Hospital Affiliated to Shandong First Medical University, Jinan 250021, China; Department of Radiology, Shandong Provincial Hospital Affiliated to Shandong First Medical University, Jinan 250021, China; Shandong University, Jinan, Shandong 250021, China; Department of Neurology, Shandong Provincial Hospital Affiliated to Shandong First Medical University, Jinan 250021, China; Department of Neurology, Shandong Provincial Hospital Affiliated to Shandong First Medical University, Jinan 250021, China; Philips Healthcare, Shanghai 200000, China; Department of Neurology, Shandong Provincial Hospital Affiliated to Shandong First Medical University, Jinan 250021, China; Department of Radiology, Shandong Provincial Hospital Affiliated to Shandong First Medical University, Jinan 250021, China

**Keywords:** glymphatic system, diffusion tensor image analysis along the perivascular space (DTI-ALPS), Parkinson’s disease, cognition, grey matter

## Abstract

The glymphatic system may play a central role in cognitive impairment associated with Parkinson's disease, but its relationship with regional cortical atrophy is not fully explored. To explore associations among glymphatic dysfunction, regional cortical degeneration and cognitive impairment in Parkinson’s disease participants, we evaluated 51 participants with documented Parkinson’s disease (28 men; age, 61.65 ± 8.27 years) and 30 age- and sex-matched normal controls (11 men; age, 59.2 ± 5.90 years) who underwent 3.0-T MRI of the brain, including high-resolution T1-weighted imaging and diffusion-tensor imaging along the perivascular space as a surrogate for glymphatic flow. Cortical grey matter volume was segmented automatically based on three-dimensional T1-weighted sequences. Cognitive function was assessed by Mini-Mental State Examination. The relationship between glymphatic dysfunction, cognitive decline and regional cortical degeneration was explored. The participants with Parkinson’s disease revealed lower diffusion-tensor imaging along the perivascular space (1.45 ± 0.17 versus 1.64 ± 0.17, *P* < 0.0001) as compared with normal controls, indicating disturbed glymphatic flow. Glymphatic dysfunction was associated with cognitive scores (*r* = 0.54, *P* = 0.003). Diffusion-tensor imaging along the perivascular space values were positively associated with the volume of specific cortical regions (all *P*-values <0.05) including the temporal pole, posterior orbital gyrus, orbital part of the inferior frontal gyrus, frontal operculum, central operculum and anterior cingulate gyrus. Mediation analysis within the Parkinson’s disease participants indicated that the relationship between glymphatic dysfunction and cognitive scores was partially mediated by the integrity of orbital part of the inferior frontal gyrus and anterior cingulate gyrus. Glymphatic dysfunction is associated with cognitive decline in Parkinson’s disease, whereas the distribution of regional cortical degeneration may constitute the link between glymphatic dysfunction and cognitive impairment.

## Teaser

Yang *et al*. report that glymphatic dysfunction is associated with cognitive decline in patients with Parkinson's disease. The study finds that the relationship between glymphatic dysfunction and cognitive impairment in Parkinson’s disease group is mediated by degeneration in specific cortical regions, including the inferior frontal gyrus and anterior cingulate gyrus.

## Graphical Abstract

**graphical abstract:**
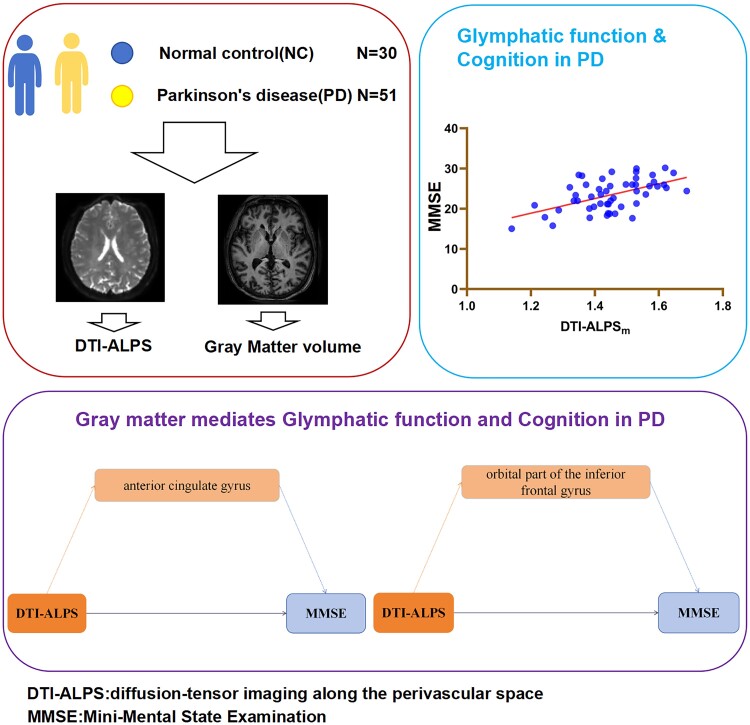

## Introduction

Cognitive impairment is one of the most prevalent and incapacitating non-motor symptoms of Parkinson's disease.^1,2^ Approximately 40% of patients with Parkinson’s disease will suffer from cognitive impairment, increasing the risk of developing dementia.^3^ Cognitive impairment in Parkinson’s disease may be associated with cortical degeneration, potentially stemming from the accumulation of ɑ-synuclein pathology in susceptible cortical regions.^4-6^ However, the typical pattern of cortical degeneration in Parkinson's disease has not been fully established as well as the underlying mechanisms remain incompletely understood.

Recently, a highly organized fluid-clearance pathway known as the glymphatic system has been attracting increasing attention.^7^ The glymphatic system constitutes a comprehensive cerebral fluid transport network that facilitates the removal of neurotoxic metabolic byproducts such as amyloid-β and tau from the brain parenchyma by promoting the drainage of cerebrospinal fluid along the perivascular space.^8-10^ Previous investigations have shown that deterioration in the glymphatic pathway could exacerbate ɑ-synuclein pathology and subsequently lead to the loss of neurons in Parkinson’s disease.^11,12^ Therefore, we hypothesize that dysfunction of the glymphatic system may be associated with regional cortical atrophy and may indirectly contribute to cognitive decline in patients with Parkinson’s disease.

A novel method, the diffusion tensor image analysis along the perivascular space (DTI-ALPS), has been proposed to assess glymphatic function.^13,14^ Previous investigations have noted a lower DTI-ALPS index in the patients with Parkinson’s disease compared with normal control subjects.^15^ To the best of our knowledge, the potential link between glymphatic dysfunction and regional cortical atrophy in patients with Parkinson’s disease has not been fully explored. Accordingly, the purpose of our study was to explore the relationship among global glymphatic function assessed by DTI-ALPS, cognition and the spatial distribution of cortical degeneration in patients with Parkinson’s disease.

## Materials and methods

### Participants

**Figure 1:**
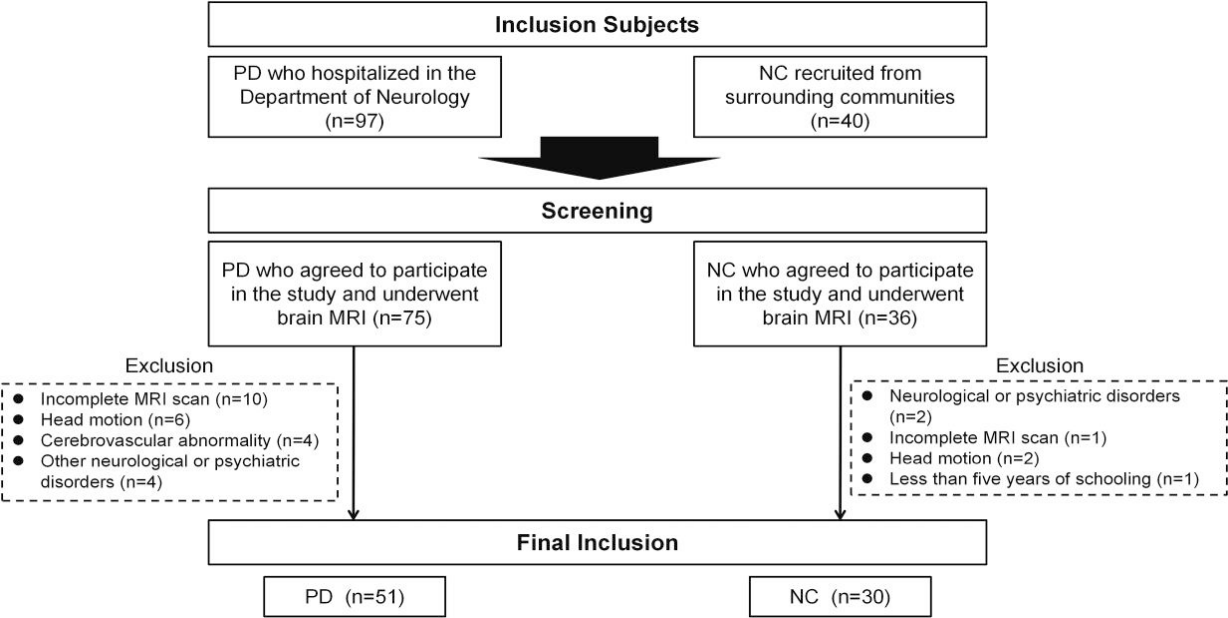
**Flow diagram of the study sample.** PD, Parkinson's disease; NC, normal control.

Participants were recruited between January 2021 and May 2024 from Shandong Provincial Hospital, which is affiliated with Shandong First Medical University, and from nearby communities (Fig. 1). This study received ethical approval from the local ethics committee, and informed consent was obtained from all participants. Patients were diagnosed with the idiopathic form of Parkinson's disease by neurologists according to the criteria of the UK Parkinson's Disease Society Brain Bank.^2^ For normal controls, individuals had to have normal subjective cognition, and they had to be at least 40 years old with at least 5 years of formal schooling. Participants were excluded from the study if they had (i) other neurological or psychiatric disorders, such as stroke, epilepsy, uncontrolled depression or brain tumors, (ii) Cerebrovascular abnormality, (iii) incomplete MRI scan or low image quality. Cognitive performance was evaluated in all individuals with Parkinson's disease using the Mini-Mental State Examination (MMSE).

### Image acquisition

Patients and controls underwent magnetic resonance imaging on a 3.0-T Ingenia CX scanner (Philips Healthcare, Best, the Netherlands) equipped with a 32-channel head coil. Diffusion tensor imaging was performed using a single-shot spin-echo echo-planar imaging sequence with the following parameters: number of axial slices, 66; field of view, 224 × 224 × 132 mm^3^; TR (repetition time), 2726 ms; TE (echo time), 76 ms; flip angle, 90 °; voxel size, 2.0 × 2.0 × 2.0 mm^3^; number of gradient directions, 128; and b, 1000 s/mm^2^. High-resolution T1-weighted imaging was performed with MPRAGE (Magnetization Prepared Rapid Acquisition Gradient Echo) sequence and the following parameters: number of sagittal slices, 160; field of view, 256 × 256 × 160 mm^3^; TR, 2726 ms; TE, 76 ms; TI (inversion time), 950 ms; flip angle, 8°; and voxel size, 1.0 × 1.0 × 1.0 mm^3^. Imaging of each participant required a total of 10 min.

### Image quality control

Image quality was evaluated using both subjective and objective approaches. Subjective assessment involved visual inspection by experienced radiologists for the presence of artefacts, anatomical integrity and overall image clarity. Next, structural image quality was assessed using the image quality rate from Computational Anatomy Toolbox 12.7 (http://dbm.neuro.uni-jena.de/cat) within the Statistical Parameter Mapping 12 package (http://www.fil.ion.ucl.ac.uk/spm) to ensure objective evaluation. To prevent the underestimation of grey matter in preprocessing, subjects with an image quality rate below B- were excluded, following strict quality control protocols.

### Image processing

Diffusion tensor imaging data were pre-processed using FSL 6.0.1 (FMRIB Software Library, www.fmrib.ox.ac.uk/fsl), during which top-up and eddy current correction were applied. Images were then reconstructed using the Q-space diffeomorphic reconstruction method^16^ in DSI STUDIO (http://dsi-studio.labsolver.org), during which the images were transformed into the standard Montreal Neurological Institute space and normalized to the ‘ICBM152_adult’ atlas. Four regions of interest were manually defined in bilateral areas of interest, and the regions included association fiber and projection fiber at the level of the lateral ventricle body in order to permit calculation of the diffusivity values Dxx, Dyy and Dzz. The DTI-ALPS index of glymphatic function was calculated separately in the left and right hemispheres using the formula:


$$\begin{array}{rl} \mathrm{DTI}\text{-}\mathrm{ALPS}\;\mathrm{index}\;= & \;\mathrm{mean}\;\mathrm{of}\;\mathrm{Dx}x_{\mathrm{proj}}\;\mathrm{and}\\ & \;\mathrm{Dx}x_{\mathrm{assoc}}/\mathrm{mean}\;\mathrm{of}\;\mathrm{Dy}y_{\mathrm{proj}}\;\mathrm{and}\;\mathrm{Dz}z_{\mathrm{assoc}} \end{array}$$

The researcher calculating the DTI-ALPS values was blinded to the clinical data to prevent potential bias. To assess the reproducibility of the DTI-ALPS index, the region of interest (ROI) was repeatedly placed by the same radiologist (Y.Z.) for each participant. After 3 months, we obtained DTI-ALPS values and calculated intraclass correlation coefficients.

T1-weighted imaging data were analyzed using the CAT12 software. Images were segmented into grey matter, white matter, and cerebrospinal fluid using the default pre-processing pipeline in the Toolbox, including intensity normalization, biased field correction and transformed into the Montreal Neurological Institute template. Grey matter volume in each region of interest, as defined in the neuromorphometric atlas of the Toolbox, was calculated using the function ‘Estimate mean values inside ROI’. Grey matter volume for bilateral brain structures was obtained by summing the volumes for each hemisphere (Supplementary Fig. 1).

### Statistical analysis

Statistical analysis was conducted using SPSS 26 (IBM SPSS, Armonk, NY, USA) and GraphPad Prism software (v9, GraphPad Inc., San Diego, CA, USA). Inter-group differences in continuous variables were evaluated using Analysis of Covariance while controlling for the potential confounding effects of age and sex if the data were normally distributed based on the Shapiro–Wilk test; otherwise, the quade test was utilized. Inter-group differences in categorical variables were assessed using the χ^2^ test.

Partial correlation analysis was used to detect three types of associations in Parkinson’s disease group: (1) between DTI-ALPS index and cognitive performance, (2) between DTI-ALPS index and grey matter volume, and (3) between grey matter volume and cognitive performance. Analysis 1 was adjusted for age, sex and education, analysis 2 was adjusted for age, sex and total intracranial volume, and analysis 3 was adjusted for age, sex, education and total intracranial volume. The ability of grey matter volume to mediate an observed association between DTI-ALPS and cognitive performance was assessed using Preacher and Hayes analysis.^17^ Differences with *P* < 0.05 were considered statistically significant. The Benjamini–Hochberg method was used to correct for multiple comparisons.

## Results

### Demographic data

The study encompassed 51 individuals (28 men; age, 61.65 ± 8.27 years) diagnosed with Parkinson's disease and 30 healthy controls (11 men; age, 59.2 ± 5.90 years) with normal cognitive function. The patients with Parkinson's disease did not differ significantly in age, sex distribution or other demographic characteristics from the cognitively normal controls in our study (Table 1). The 51 patients in the Parkinson's disease group had left-sided onset in 23 cases and right-sided onset in 28 cases. The mean disease duration was 4.08 ± 3.28 years, with a Hoehn–Yahr stage of 2.5 (range: 2–3), and a mean MMSE score of 23.9 ± 4.38. Thirty patients with Parkinson’s disease had a mean UPDRS III score of 29.60 ± 11.59.

**Table 1:** Patients demographics and neuropsychological results

	NC	Parkinson’s disease	*P* value
	*N* = 30	*N* = 51	
Age (years)	59.2 ± 5.90	61.65 ± 8.27	0.16
Sex, male, *N* (%)	11 (37%)	28 (54.9%)	0.18
Height (cm)	165.03 ± 7.16	164.30 ± 5.42	0.61
Weight (kg)	66.31 ± 11.16	64.57 ± 7.23	0.40
Education (years)	13.0 ± 3.2	12.3 ± 3.7	0.39
Symptom onset side, *N* (%)			
Left-side		23 (45%)	
Right-side		28 (55%)	
Disease duration (years)		4.08 ± 3.28	
H&Y stage		2.5 (2.3)	
MMSE scores		23.9 ± 4.38	

Values are presented as mean ± SD for quantitative variables and as frequency (percentage) for categorical variables. Group comparisons between the control and Parkinson’s disease groups were conducted using ANCOVA or the quade test for quantitative variables and the χ^2^ test for categorical variables.

### Comparison of DTI-ALPS between Parkinson’s disease participants and normal controls

**Figure 2:**
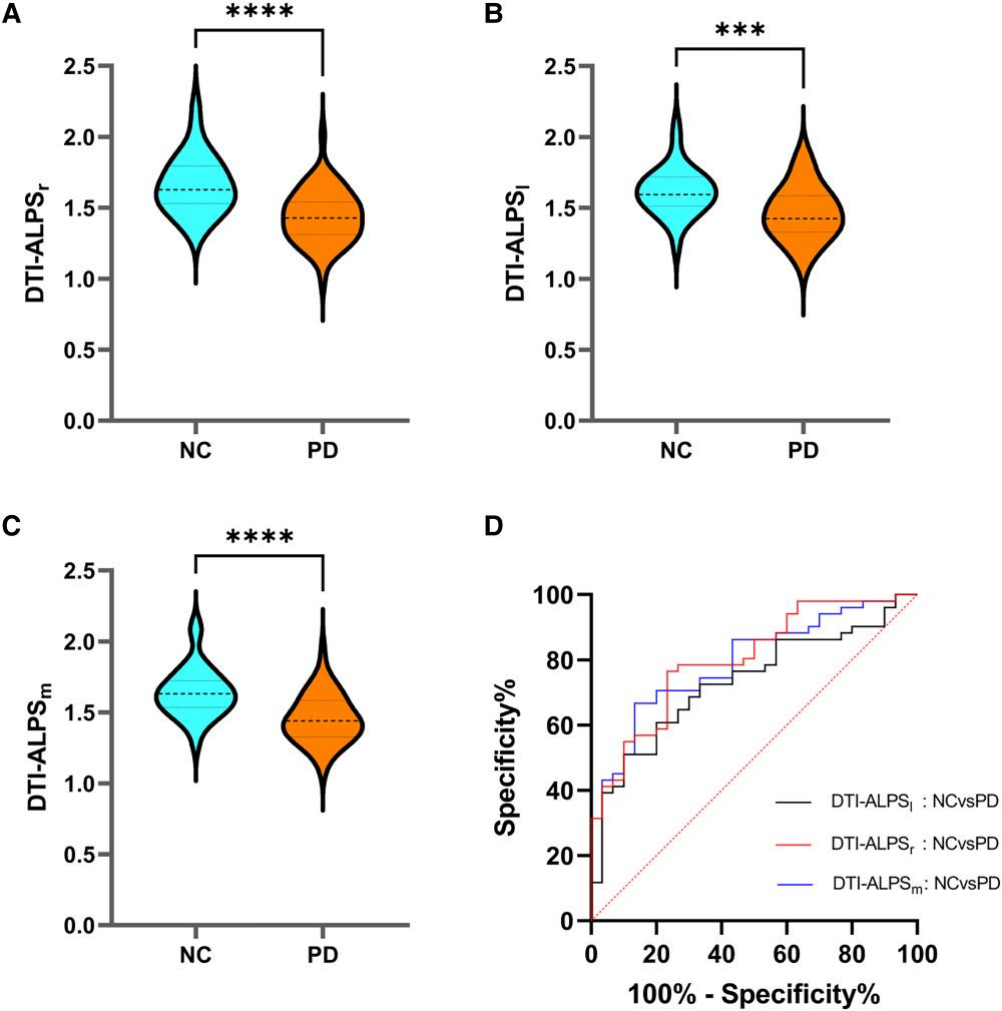
**Statistical analysis results of the diffusion tensor image analysis along the perivascular space (DTI-ALPS) indexes.** (**A–C**) Comparison of DTI-ALPS indexes between Parkinson’s disease (*n* = 51) and NC (*n* = 30) using ANCOVA (Analysis of Covariance). Statistics values for **A–C**: *F* = 24.00, *P* ＜ 0.0001; *F* = 14.14, *P* = 0.0004; *F* = 22.55, *P* ＜ 0.0001, respectively. (**D**) Receiver operating characteristic (ROC) curves for diagnosing Parkinson’s disease (*n* = 51) and NC (*n* = 30) using DTI-ALPS indexes. The AUC of DTI-ALPS_l_, DTI-ALPS_r_ or DTI-ALPS_m_ for distinguishing patients with Parkinson’s disease from NCs was 0.73 (black line), 0.80 (red line) or 0.79 (blue line), respectively. DTI-ALPS_r_, right-hemispheric diffusion tensor image analysis along the perivascular space (DTI-ALPS) indexes; DTI-ALPS_l_, left-hemispheric diffusion tensor image analysis along the perivascular space (DTI-ALPS) indexes; DTI-ALPS_m_, mean diffusion tensor image analysis along the perivascular space (DTI-ALPS) indexes; NC, normal control. ***P value <0.001; ****P value <0.0001.

The intraclass correlation coefficient defined by the ROI in this study was 0.88 (*P* < 0.001). Figure 2 and Supplementary Table 1 show the differences in the left DTI-ALPS index (DTI-ALPS_l_), the right DTI-ALPS index (DTI-ALPS_r_) and the mean DTI-ALPS index (DTI-ALPS_m_) between the Parkinson’s disease group and the normal control group. The DTI-ALPS index of patients with Parkinson’s disease was significantly lower than that of normal controls (DTI-ALPS_r_, 1.44 ± 0.18 versus 1.66 ± 0.20, *F* = 24.00, *P* < 0.0001; DTI-ALPS_l_, 1.46 ± 0.19 versus1.62 ± 0.17, *F* = 14.14, *P* = 0.0004; DTI-ALPS_m_, 1.45 ± 0.17 versus 1.64 ± 0.17, *F* = 22.55, *P* < 0.0001).

Based on receiver operating characteristics curve, the AUC of DTI-ALPS_l_, DTI-ALPS_r_ or DTI-ALPS_m_ for distinguishing patients with Parkinson’s disease from NCs was 0.73, 0.80 or 0.79, respectively.

### Correlations of DTI-ALPS indexes with neuropsychological tests in Parkinson’s disease

**Figure 3:**
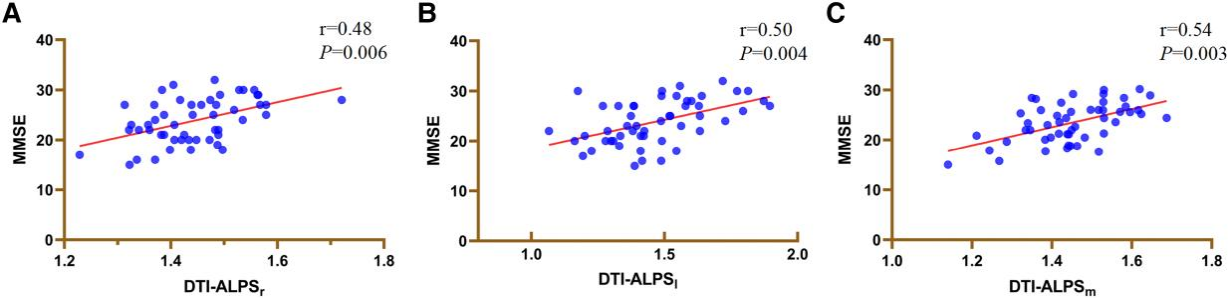
**The relationship between DTI-ALPS and cognitive function.** (**A–C**) Partial correlation analysis between DTI-ALPS indexes and neuropsychological tests after correction for age, sex and education years in Parkinson's disease group (*n* = 51). Each data point represents an individual patient with Parkinson's disease. DTI-ALPS_r_: right-hemispheric diffusion tensor image analysis along the perivascular space (DTI-ALPS) indexes. DTI-ALPS_l_, left-hemispheric diffusion tensor image analysis along the perivascular space (DTI-ALPS) indexes; DTI-ALPS_m_, mean diffusion tensor image analysis along the perivascular space (DTI-ALPS) indexes; MMSE, Mini-Mental State Examination. ****P value <0.0001.

Partial correlation analysis between the MRI indexes and neuropsychological tests was performed in all patients with Parkinson’s disease. DTI-ALPS index correlated positively with MMSE score in the Parkinson’s disease group (DTI-ALPS_l,_*r* = 0.50, *P* = 0.004; DTI-ALPS_r_, *r* = 0.48, *P* = 0.006 DTI-ALPS_m_, *r* = 0.54, *P* = 0.003) after adjustment for age, sex and educational years (Fig. 3A–C, Supplementary Table 2).

### Association among DTI-ALPS, cortical grey matter volume and MMSE scores in Parkinson’s disease

**Figure 4:**
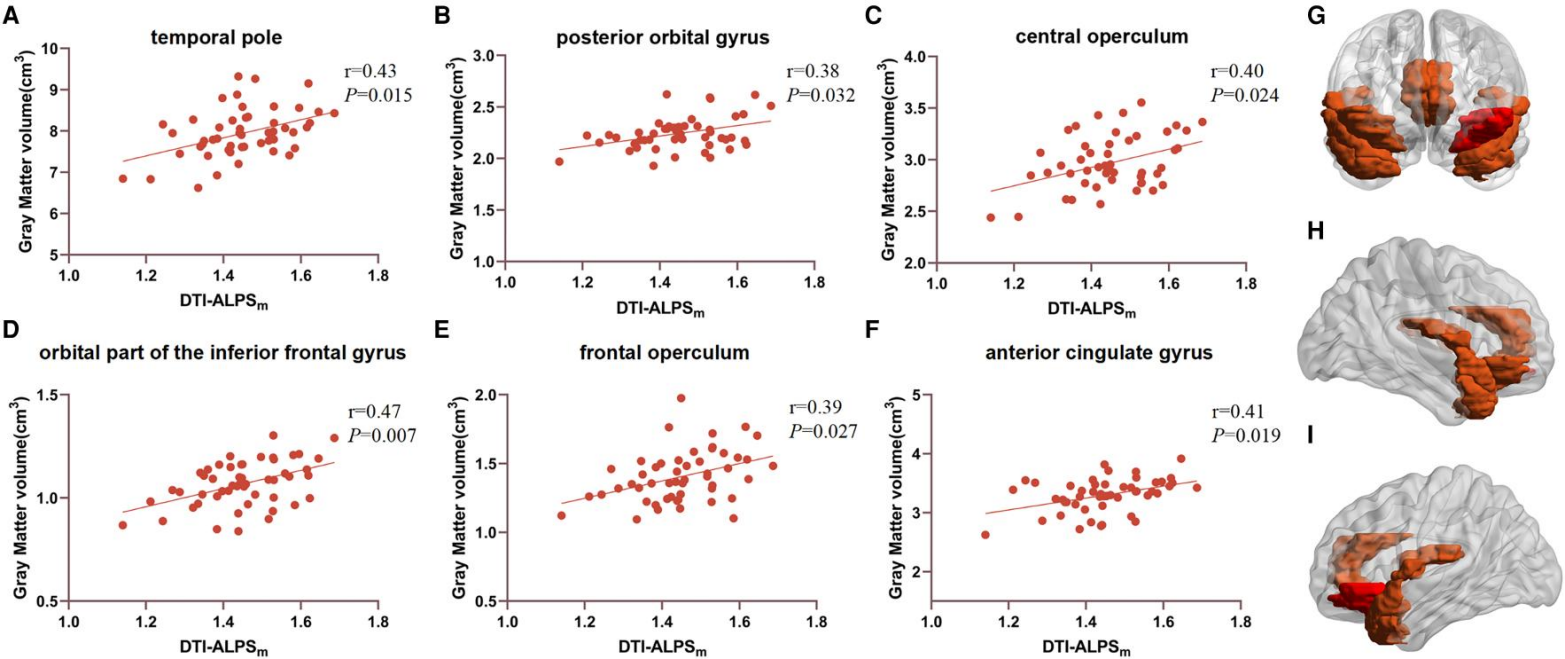
**The relationship between DTI-ALPS and grey matter volume.** (**A–F**) Partial correlation analysis between DTI-ALPS_m_ indexes and grey matter volume (cm^3^) in different regions of the brain after correction for age, sex and total intracranial volume in Parkinson's disease group (*n* = 51). Each data point represents an individual patient with Parkinson's disease. (**G–I**) Brain regions (red color) showing significant correlation with DTI-ALPS_m_ in Parkinson's disease group (*n* = 51), including temporal pole, posterior orbital gyrus, orbital part of the inferior frontal gyrus, frontal operculum, central operculum, and anterior cingulate gyrus. DTI-ALPS_m_, mean diffusion tensor image analysis along the perivascular space (DTI-ALPS) indexes.

Figure 4 and Supplementary Table 3 show that several cortical regions, adjusted for age, sex and total intracranial volume, were found to be associated with the mean DTI-ALPS, including the temporal pole (*r* = 0.43, *P* = 0.015), posterior orbital gyrus (*r* = 0.38, *P* = 0.032), orbital part of the inferior frontal gyrus (*r* = 0.47, *P* = 0.007), frontal operculum (*r* = 0.39, *P* = 0.027), central operculum (*r* = 0.40, *P* = 0.024), and anterior cingulate gyrus (*r* = 0.41, *P* = 0.019).

**Figure 5:**
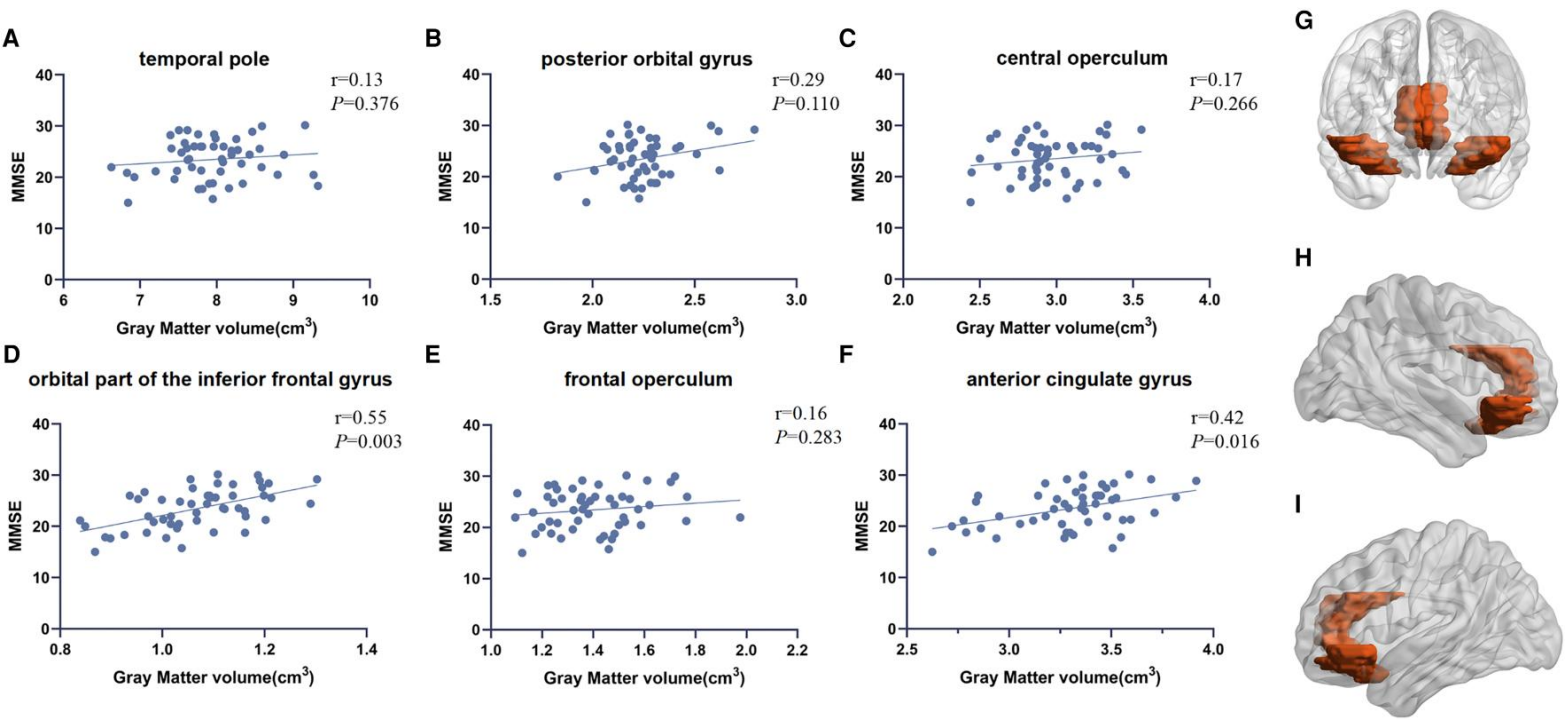
**The relationship between cognitive function and grey matter volume.** (**A–F**) Partial correlation analysis between MMSE scores and grey matter volume (cm^3^) in different regions of the brain after correction for age, sex, total intracranial volume and education years in Parkinson's disease group (*n* = 51). Each data point represents an individual patient with Parkinson's disease. (**G–I**) Brain regions (red color) showing significant correlation with MMSE in Parkinson's disease group (*n* = 51), including orbital part of the inferior frontal gyrus and anterior cingulate gyrus. MMSE, Mini-Mental State Examination.

Based on the brain regions associated with DTI-ALPS, we found that some regions were correlated with MMSE scores, adjusted for age, sex, education and total intracranial volume. The topographic maps demonstrate overlapping areas between clusters of regions exhibiting consistency with MMSE scores and those with DTI-ALPS within the Parkinson’s disease group, notably including the orbital part of the inferior frontal gyrus (*r* = 0.55, *P* = 0.003) and anterior cingulate gyrus (*r* = 0.42, *P* = 0.016) but not including the posterior orbital gyrus (*r* = 0.29, *P* = 0.110), central operculum (*r* = 0.17, *P* = 0.266), temporal pole (*r* = 0.13, *P* = 0.376) or frontal operculum (*r* = 0.16, *P* = 0.283). (Figure 5, Supplementary Table 4).

### Mediation analysis in Parkinson’s disease group showed partial mediation of grey matter volume in DTI-ALPS index and MMSE scores

**Figure 6:**
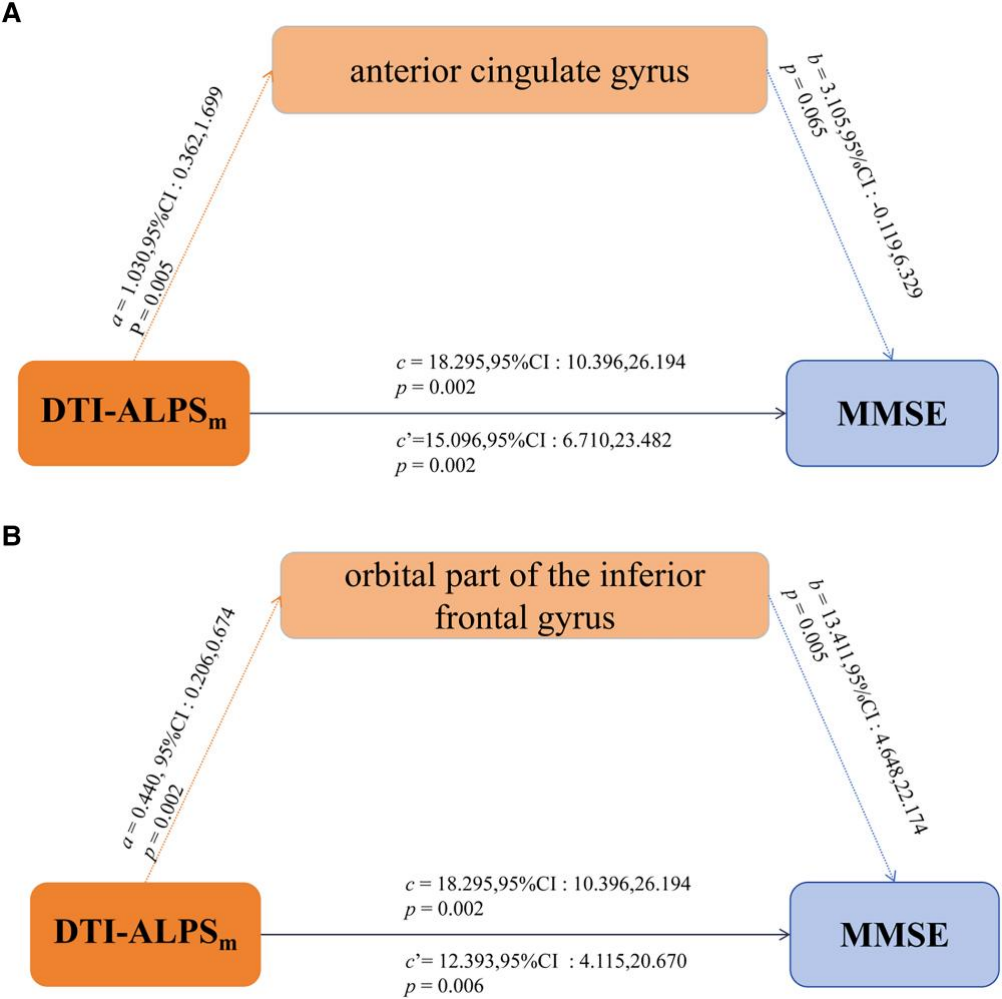
**Statistical analysis results of mediation analysis.** Mediation analysis to assess whether grey matter volume in the two indicated brain regions mediates the observed associations between DTI-ALPS_m_ index as the predictor and score on the Mental Mini-State Examination as the outcome. a, b, c, and c’ are coefficients representing unstandardized regression weights in Parkinson's disease group (*n* = 51). The c path coefficient refers to the total effect of DTI-ALPS_m_ index on MMSE scores. The c’ path coefficient refers to the direct effect of the DTI-ALPS_m_ index on scores. CI, confidence interval; DTI-ALPS_m_, mean diffusion tensor image analysis along the perivascular space (DTI-ALPS) indexes; MMSE, Mini-Mental State Examination.

Considering the correlation between DTI-ALPS and MMSE scores, we conducted tests to examine whether this relationship could potentially be mediated by the documented loss of grey matter in different brain regions. We found that grey matter volume in the anterior cingulate gyrus and orbital part of the inferior frontal gyrus could partially mediate the observed association between DTI-ALPS index and MMSE score, when we controlled for age, sex, education and total intracranial volume. The mediated effects were 17.49% and 32.24% in the anterior cingulate gyrus and orbital part of the inferior frontal gyrus, respectively (Fig. 6, Supplementary Table 5).

## Discussion

In this study, we used the DTI-ALPS index as a surrogate for glymphatic clearance function to investigate the relationship between glymphatic dysfunction, regional cortical degeneration and cognitive decline in patients with Parkinson's disease. Our analysis revealed three main findings. Firstly, we found a lower DTI-ALPS index within the Parkinson group compared with that value in normal subjects, indicating glymphatic dysfunction in Parkinson patients. Of note, this reduction in glymphatic function was associated with the severity of cognitive impairment in individuals with Parkinson's disease. Secondly, we identified several specific cortical regions that showed correlations with the DTI-ALPS index within the Parkinson group, suggesting the presence of regional vulnerabilities associated with glymphatic dysfunction. Thirdly, our findings suggest that specific cortical areas related to DTI-ALPS indices modulate the severity of glymphatic clearance impairment on cognitive decline. These observations may indicate that glymphatic activity is not directly associated with cognitive function in Parkinson's disease but may indirectly be modulated by the integrity of specific cortical regions.

Our study found that individuals with Parkinson's disease had a significantly lower DTI-ALPS index than cognitively normal controls. This finding aligns with previous research and suggests a potential involvement of glymphatic dysfunction in the pathogenesis of Parkinson's disease.^15,18-24^

This study revealed a positive correlation between the DTI-ALPS indices and MMSE scores in Parkinson's disease, indicating a robust relationship between glymphatic clearance function and cognitive performance. The disruption of ɑ-synuclein clearance may be responsible for this phenomenon, leading to its aggregation, increased cytotoxicity and more rapid cognitive decline. Ongoing investigation aims to elucidate how glymphatic dysfunction contributes to ɑ-synuclein aggregation in Parkinson's disease. Recent findings have shown that aquaporin-4 (AQP4) deficiency in mice exacerbates alpha-synuclein pathology in Parkinson’s disease animal models. AQP4, predominantly situated in the perivascular astrocyte end-feet, functions as a pivotal element of the glymphatic system.^25^ In mice overexpressing human A53T-ɑ-synuclein, the absence of AQP4 hastened the accumulation of ɑ-synuclein, exacerbated the depletion of dopaminergic neurons and accelerated the manifestation of Parkinson's disease-like symptoms.^26^ Furthermore, the upregulation of A53T-ɑ-synuclein decreased AQP4 expression and inhibited glymphatic activity in mice, thereby establishing a detrimental feedback loop.

Furthermore, we explored how glymphatic impairment relates to regional cortical degeneration, providing some potential clues about potential links between brain waste clearance and neurodegenerative processes. Our work identified several cortical regions that are strongly associated with the DTI-ALPS indices, indicating that glymphatic impairment may manifest as regional vulnerability. In patients with idiopathic normal-pressure hydrocephalus, gadolinium injection resulted in the regional enhancement of brain parenchyma in various areas, including the pons, thalamus, periventricular frontal horn, inferior frontal gyrus and precentral gyrus, suggesting a specific distribution pattern that may result in regional brain toxicity.^27^ Interestingly, the tracer did not distribute freely in the brain but instead traveled along the three main cerebral arteries (anterior, middle and posterior cerebral arteries). Moreover, the gadolinium-based contrast agent spread further in a forward direction, suggesting that glymphatic activity varied among patients and brain regions.^28^ Braak proposed a typical progression pattern of ɑ-synuclein accumulation in Parkinson's disease, which propagates initially through the brainstem, then enters limbic structures, and ultimately spreads to the neocortex.^29^ This theory highlights the spatially and temporally heterogeneous nature of ɑ-synuclein distribution. Our study showed DTI-ALPS values were positively associated with the volume of specific areas in the Parkinson’s disease group, which partially overlapped with the brain regions where ɑ-synuclein accumulates. It has been suggested that the pattern of ɑ-synuclein aggregate formation in Parkinson's disease closely mirrors the pattern of contrast agent distribution in the brain parenchyma following intrathecal injection.^30^ Consequently, we speculate that the degeneration of these regions is initiated by the effects of neurotoxic proteins resulting from impaired glymphatic activity.

Prior research has suggested that cognitive impairment in Parkinson’s disease is neurologically defined by the accumulation of ɑ-synuclein in the cortex, encompassing the limbic and neocortical regions of the brain.^6,31^ Given the significant correlations found between DTI-ALPS and cognitive function, we investigated whether this association was mediated by cortical integrity. Mediation analysis revealed that the orbital part of the inferior frontal gyrus and anterior cingulate gyrus may mediate the observed relationship between DTI-ALPS and global cognitive performance. This observation suggests that the integrity of specific grey matter regions plays a more direct role in cognitive decline than the dysfunction of the glymphatic clearance. The stagnation of glymphatic flow may contribute to protein aggregation, resulting in neuronal loss and ultimately leading to dementia. Both the anterior cingulate gyrus and orbital part of the inferior frontal gyrus play key roles in higher-order cognitive processes, such as decision-making, emotion regulation and executive function. These regions are part of the prefrontal cortex, known for its vulnerability in neurodegenerative disorders, including Parkinson’s disease. Specifically, the anterior cingulate gyrus is extensively involved in cognitive, sensation^32,33^ and sleep disorders.^34^ Previous studies have indicated that the orbital part of the inferior frontal gyrus, an essential component of the prefrontal cortex, significantly influences cognition and language processing.^35-37^

Several limitations are inherent in this study. Firstly, this cross-sectional study only used the baseline dataset to assess glymphatic function and their effects on specific brain regions, longitudinal studies with larger samples are needed in future. Secondly, this work did not explore how declines in glymphatic function, grey matter volume and cognitive performance interact during the process of normal aging, which could be crucial for developing interventions aimed at attenuating cognitive decline, even among individuals without neurodegenerative disorders. Third, in addition to pathophysiological changes in patients with Parkinson's disease, certain factors—such as sleep quality, alcohol history, and white matter injury—may influence glymphatic function. The absence of these data may limit our ability to fully assess glymphatic activity. Lastly, the correlation between the DTI-ALPS and human glymphatic function has not been rigorously validated by pathophysiological studies, so its relationship with glymphatic clearance should be interpreted cautiously. Additionally, DTI-ALPS was also influenced by other factors, such as white matter microstructure, direct comparisons between glymphatic activity measurements across different imaging modalities and diffusion MRI methods are still needed in the future.

Despite its limitations, our work provides evidence linking glymphatic dysfunction to cortical degeneration and cognitive impairment in Parkinson's disease. Preliminary evidence suggests that the impact of glymphatic function on cognitive performance is not direct but rather indirect, mediated through its clearance function to maintain the integrity of the cerebral cortex. The monitoring of glymphatic activity and its subsequent impact on cortical degeneration holds promise for monitoring neurodegenerative conditions like Parkinson's disease.

## Supplementary Material

fcaf029_Supplementary_Data

## Data Availability

The data that support the findings of this study are available from the corresponding author upon reasonable request.
