# Supplementary material for: Glymphatic dysfunction exacerbates cognitive decline by triggering cortical degeneration in Parkinson's disease: evidence from diffusion-tensor MRI
Source: Brain Commun. 2025 Feb 20;7(1):fcaf029. doi: 10.1093/braincomms/fcaf029 (PMC11840164; doi:10.1093/braincomms/fcaf029)
Supplement: fcaf029_Supplementary_Data [file fcaf029_supplementary_data.docx]

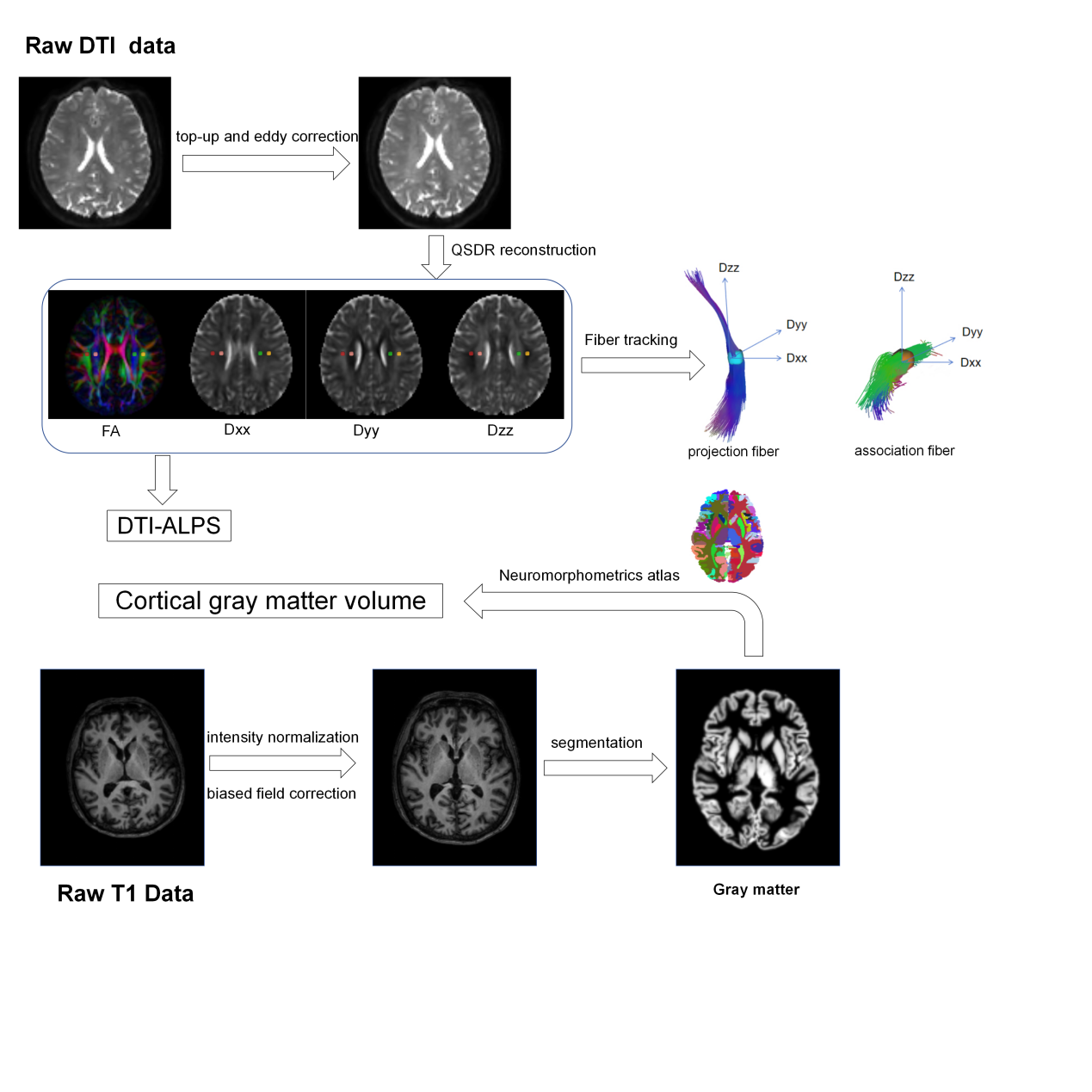


**Supplementary Figure 1: DTI-ALPS and cortical gray matter volume calculation method.**

DTI images were preprocessed by using topup and eddy current, reconstructed by using Q-Space Diffeomorphic reconstruction (QSDR) method. Fractional anisotropy (FA) color map, and the diffusivity in the directions of x-axis (Dxx), y-axis (Dyy), and z-axis (Dzz) on each image were created. The ROIs were drawn in the FA maps and then transferred to the Dxx, Dyy, and Dzz maps.

For morphometric analysis of the imaging data, the default processing pipeline was applied, which includes bias correction, intensity normalization, and segmentation into gray matter. Gray matter volume estimates were extracted from the left and right hemispheres for all regions of interest (ROI) of the Neuromorphometrics atlas.

|  | NC | PD | F | *P* value | FDR*P* |
| --- | --- | --- | --- | --- | --- |
|  | n=30 | n=51 |  |  |  |
| DTI-ALPS_m_ | 1.64±0.17 | 1.45±0.17 | 22.55 | ＜0.0001**** | ＜0.0001**** |
| DTI-ALPS_r_ | 1.66±0.20 | 1.44±0.18 | 24.00 | ＜0.0001**** | ＜0.0001**** |
| DTI-ALPS_l_ | 1.62±0.17 | 1.46±0.19 | 14.14 | 0.0003*** | 0.0004*** |

**Supplementary Table 1: Comparison of DTI-ALPS indexes between PD (n=51) and NC (n=30) using ANCOVA (Analysis of Covariance).**

DTI-ALPS_r_: right-hemispheric diffusion tensor image analysis along the perivascular space (DTI-ALPS) indexes. DTI-ALPS_l_: left-hemispheric diffusion tensor image analysis along the perivascular space (DTI-ALPS) indexes. DTI-ALPS_m_: mean diffusion tensor image analysis along the perivascular space (DTI-ALPS) indexes. FDR:false discovery rate ****indicates *P* value < 0.0001 ***indicates *P* value < 0.001.

|  |  | MMSE | | |
| --- | --- | --- | --- | --- |
| Parameter | Value | r | *P* | FDR *P* |
| DTI-ALPS_l_ | 1.46±0.19 | 0.50 | 0.0002*** | 0.004** |
| DTI-ALPS_r_ | 1.44±0.18 | 0.48 | 0.0004*** | 0.006** |
| DTI-ALPS_m_ | 1.45±0.17 | 0.54 | 0.0001*** | 0.003** |

**Supplementary Table 2: Partial correlation analysis between DTI-ALPS indexes and neuropsychological tests after correction for Age, Sex and Education Years in Parkinson’s disease group (n=51).**

DTI-ALPS_r_: right-hemispheric diffusion tensor image analysis along the perivascular space (DTI-ALPS) indexes. DTI-ALPS_l_: left-hemispheric diffusion tensor image analysis along the perivascular space (DTI-ALPS) indexes. DTI-ALPS_m_: mean diffusion tensor image analysis along the perivascular space (DTI-ALPS) indexes.MMSE：Mini-Mental State Examination FDR:false discovery rate ***indicates *P* value < 0.001 **indicates *P* value < 0.01.

|  |  | DTI-ALPS_m_ | | | MMSE | | |
| --- | --- | --- | --- | --- | --- | --- | --- |
| Region | Volume (cm^3^) | r | *P* | FDR*P* | r | *P* | FDR*P* |
| temporal pole | 7.95±0.58 | 0.43 | 0.002** | 0.015* | 0.13 | 0.3625 | 0.376 |
| posterior orbital gyrus | 2.24±0.15 | 0.38 | 0.006** | 0.032* | 0.29 | 0.0399 | 0.110 |
| orbital part of the inferior frontal gyrus | 1.06±0.11 | 0.47 | 0.0006*** | 0.007** | 0.55 | 0.0001*** | 0.003** |
| frontal operculum | 1.41±0.18 | 0.39 | 0.0048** | 0.027* | 0.16 | 0.2683 | 0.283 |
| central operculum | 2.97±0.25 | 0.40 | 0.0039** | 0.024* | 0.17 | 0.2467 | 0.266 |
| anterior cingulate gyrus | 3.30±0.28 | 0.41 | 0.0027** | 0.019* | 0.42 | 0.002** | 0.016* |

**Supplementary Table 3:** Partial correlation analysis between DTI-ALPS indexes and gray matter volume (cm^3^) in different regions of the brain after correction for Age, Sex and Total Intracranial Volume in Parkinson’s disease group (n=51) and partial correlation analysis between MMSE scores and gray matter volume (cm^3^) in different regions of the brain after correction for Age, Sex, Total Intracranial Volume and Education Years in Parkinson’s disease group (n=51) DTI-ALPS_m_: mean diffusion tensor image analysis along the perivascular; MMSE: Mini-Mental State Examination FDR:false discovery rate. ***indicates *P* value < 0.001 **indicates*P* value < 0.01 *indicates *P* value < 0.05

| Route | Coefficient | Effect | 95% CI | | SE | T | *P* | FDR *P* |
| --- | --- | --- | --- | --- | --- | --- | --- | --- |
| DTI-ALPS_m_=>anterior cingulate gyrus=>MMSE | a*b | 3.199 | 0.004 | 0.248 | 0.064 | 50.100 | 0.001** | 0.002** |
| DTI-ALPS_m_=> anterior cingulate gyrus | a | 1.030 | 0.362 | 1.699 | 0.341 | 3.020 | 0.004** | 0.005** |
| anterior cingulate gyrus=>MMSE | b | 3.105 | -0.119 | 6.329 | 1.645 | 1.888 | 0.065 | 0.065 |
| DTI-ALPS_m_=>MMSE | c' | 15.096 | 6.710 | 23.482 | 4.279 | 3.528 | 0.001** | 0.002** |
| DTI-ALPS_m_=>MMSE | c | 18.295 | 10.396 | 26.194 | 4.030 | 4.539 | 0.001** | 0.002** |

**Supplementary Table 4:** Mediation analysis to assess whether anterior cingulate gyrus volume mediates the observed associations between DTI-ALPS_m_ index as the predictor and score on the Mental Mini-State Examination as the outcome. a, b, c, and c’ are coefficients representing unstandardized regression weights. The c path coefficient refers to the total effect of DTI-ALPS_m_ index on MMSE scores. The c’ path coefficient refers to the direct effect of the DTI-ALPS_m_ index on scores. CI: confidence interval. DTI-ALPS_m_: mean diffusion tensor image analysis along the perivascular; MMSE: Mini-Mental State Examination.SE:Standard Error. **indicates *P* value < 0.01 *indicates *P* value < 0.05

| Route | Coefficient | Effect | 95% CI | | SE | t | *P* | FDR*P* |
| --- | --- | --- | --- | --- | --- | --- | --- | --- |
| DTI-ALPS_m_=>orbital part of the inferior frontal gyrus=>MMSE | a*b | 5.902 | 0.053 | 0.326 | 0.070 | 84.806 | 0.001** | 0.002** |
| ALPS=> orbital part of the inferior frontal gyrus | a | 0.440 | 0.206 | 0.674 | 0.119 | 3.686 | 0.001** | 0.002** |
| orbital part of the inferior frontal gyrus=>MMSE | b | 13.411 | 4.648 | 22.174 | 4.471 | 2.999 | 0.004** | 0.005** |
| DTI-ALPS_m_=>MMSE | c' | 12.393 | 4.115 | 20.670 | 4.223 | 2.934 | 0.005** | 0.006** |
| DTI-ALPS_m_=>MMSE | c | 18.295 | 10.396 | 26.194 | 4.030 | 4.539 | 0.001** | 0.002** |

**Supplementary Table 5:** Mediation analysis to assess whether orbital part of the inferior frontal gyrus mediates the observed associations between DTI-ALPS_m_ index as the predictor and score on the Mental Mini-State Examination as the outcome. a, b, c, and c’ are coefficients representing unstandardized regression weights. The c path coefficient refers to the total effect of DTI-ALPS_m_ index on MMSE scores. The c’ path coefficient refers to the direct effect of the DTI-ALPS_m_ index on scores. CI: confidence interval. DTI-ALPS_m_: diffusion tensor image analysis along the perivascular; MMSE: Mini-Mental State Examination.SE:Standard Error. **indicates *P* value < 0.01
